# Coenhabiting Interpersonal Inter-Identities in Recurrent Social Interaction

**DOI:** 10.3389/fpsyg.2020.00577

**Published:** 2020-03-31

**Authors:** Mark M. James, Juan Manuel Loaiza

**Affiliations:** ^1^School of Computer Science, University College Dublin, Dublin, Ireland; ^2^Independent Researcher, Newcastle Upon Tyne, United Kingdom

**Keywords:** enaction, interaction, identity, habit, enhabiting, coenhabiting, interidentity, individuation

## Abstract

We propose a view of identity beyond the individual in what we call interpersonal inter-identities (IIIs). Within this approach, IIIs comprise collections of entangled stabilities that emerge in recurrent social interaction and manifest for those who instantiate them as relatively invariant though ever-evolving patterns of being (or more accurately, becoming) together. Herein, we consider the processes responsible for the emergence of these IIIs from the perspective of an enactive cognitive science. Our proposal hinges primarily on the development of two related notions: enhabiting and coenhabiting. First, we introduce the notion of enhabiting, a set of processes at the individual level whereby structural interdependencies stabilize and thereafter undergird the habits, networks of habits, and personal identities through which we make sense of our experience. Articulating this position we lean on the notion of a tendency toward an optimal grip, though offering it a developmental framing, whereby iterative states of selective openness help realize relatively stable autonomous personal identities with their own norms of self-regulation. We then extend many of the notions found applicable here to an account of social coenhabiting, in particular, we introduce the notion of tending toward a co-optimal grip as central to the development of social habits, networks of habits, and ultimately IIIs. Such structures, we propose, also emerge as autonomous structures with their own norms of self-regulation. We wind down our account with some reflections on the implications of these structures outside of the interactions wherein they come into being and offer some thoughts about the complex animations of the individual embodied subjects that instantiate them.

We are all lichens.

Gilbert et al. (2012, p. 336)

Others, then, exist inside us, side by side with the person we are to ourselves.

Knausgaard (2017, p. 106)

Do I contradict myself? Very well then, I contradict myself.

I am large, I contain multitudes.

Whitman (2001, p. 53)

## Introduction

Interpersonal relationships have a certain stickiness to them. With help from some observations of social life, in this article, we probe into this stickiness to unravel its underlying dynamics. Imagine being in the company of an old friend and how you might experience ‘falling into’ particular patterns of being together when in their presence (Fuchs, 2017a). Without any conscious effort you take up an accent, gestures, entire patterns of being you have not wielded since you last met. The relative ubiquity of such experiences invites us to attribute some characteristics to the patterns themselves, e.g., that they have a relatively invariant identity over time (maybe recognizable in a particular accent); that they somehow transcend us as individuals, seeming to unfold with an autonomy of their own (neither of you have used *those* words since you last met, and yet you cannot seem to help yourselves). We propose to look at these patterns through the lens of an enactive approach to mind and present a conceptualization of the emergence of relatively invariant patterns in interpersonal relationships in terms of the individuation and enactment of interpersonal inter-identities (IIIs). We develop this concept in a way that expands the core enactive idea of autonomous self-production, whilst attempting to do some justice to the messy complexity and heteronomy of human social life.

Thomas Fuchs suggests the experience of ‘falling into’ a particular way of being when with a particular friend is dependent upon a dyadic body memory (Fuchs, 2017a, p. 339). This dyadic body memory, we claim, can be profitably illuminated and expanded in terms of an enactive account of the dialectics of autonomy, and the individuation of nested habits and networks of habits at multiple timescales that both organize and are organized by human social interaction. Such inter-bodily habits, goes the claim, arise within the dynamics of prolonged and/or recurrent social interactions through processes of *coenhabiting*: tending toward a *co-optimal grip* with respect to compatible concerns at multiple timescales, patterns of being together stabilize as autonomous socio-cultural structures, embedding in those that instantiate them IIIs, and thereafter shaping how they make sense together. Given the myriad social relationships available to us, we each play host to a multitude of IIIs, and given the relative autonomy of such identities and how their norms of self-regulation constrain the sense-making of their hosts, any embodied subject can be said to be partially animated by the identities it works to sustain within a given situation. Lived through by the myriad of personal and interpersonal inter-identities we help comprise, we are, thus, multiply animated.

In what follows we consider the processes that facilitate the emergence of these IIIs from an enactive perspective. We begin by reviewing Fuchs’ notion of the dyadic body memory and how it supports the emergence of relatively invariant ways of being together. We then outline some of our reasons for moving beyond this notion, ultimately suggesting that it can be further elaborated through the enactive notion of autonomy, a move that Fuchs himself seems to endorse but does not offer any details on (2017). We then look more closely at the enactive notion of autonomy, suggesting that it should function as a set of heuristics with which to make intelligible processes that support the ongoing individuation of stable identities. Here we lean on recent developments within enaction that characterize autonomy in terms of a dialectic between processes of self-production and processes of self-distinction (Di Paolo et al., 2018). We wrap up this section by suggesting how these ideas offer a good leading off point when attempting to make intelligible the processes of individuation that characterize the socio-material domain. At this juncture, we come to the central notions of enhabiting and coenhabiting.

First, we introduce the notion of enhabiting as a set of processes at the individual level whereby structural interdependencies stabilize and thereafter support the habits and identities through which we enact our worlds. Here, we lean on the notion of a tendency toward an optimal grip (e.g., Merleau-Ponty, 1945; Dreyfus, 2002; Bruineberg and Rietveld, 2014; Kiverstein and Rietveld, 2018), though employing it within a developmental framing, whereby iterative states of selective openness help realize relatively stable self-producing personal identities. We then extend this to an account of coenhabiting, a joint process that facilitates the individuation of interpersonal inter-identities through tending toward a co-optimal grip. To make our point we consider how when recurrently coordinating together toward compatible concerns at multiple timescales, nested autonomous patterns emerge with their own self-generating norms, and which are the property of the interactive system in its socio-material milieu. We speak about the evolving webs of such patterns that characterizes any recurrent social relationship in terms of interpersonal inter-identities. In the closing section, before concluding, we point toward some corollaries of the main account: firstly, what we refer to as trans-situational concerns, i.e., the beginnings of an account of how the dynamics that underwrite the emergence of interpersonal inter-identities continue to shape various modes of individual sense-making even when apart from real-time reciprocal interactions; and secondly, we characterize the embodied subject as being multiply animated, i.e., something that not only lives through the identities it manifests in relationship with others, but is also lived through by them and the larger entities that give those identities shape, e.g., the trans-individual habitus that operate at more distributed spatiotemporal scales than the interpersonal inter-identities accounted for here (Bourdieu, 1977).

## Dyadic Body Memory and Beyond

### Dyadic Body Memory

We all have old friends or family members, with whom, when we meet, we are surprised to find ourselves acting in ways, in our speech, in our gestures and so on, that we have not done since last we met. We might say things like “something about being with you just brings it out of me.” For Thomas Fuchs, such invariances rely upon what he terms a dyadic body memory, wherein any “particular interaction, when repeated, acquires its own history, thus pre-figuring and constraining future interactions between the respective partners” (Fuchs, 2017b, p. 204). What emerges is a ‘joint procedural field,’ that preordains certain interactional dynamics, e.g., particular postures, gestures, accents, dialects, and so on. Such a field might also include relatively invariant patterns of joint acting, e.g., the action arches observed in the relationship between child and caregiver during nappy changing, wherein with repeating instances there can be observed a characteristic beginning, middle, and end to the action (Rossmanith et al., 2014)^1^. Under such conditions, one often has the feeling of falling into patterns of acting, characterized by what Fuchs refers to as a kind of “unintentional entrainment” (Fuchs, 2017a, p. 339).

Fuchs employs the example of a pair of dancers to illustrate how such a form of memory serves the dyadic system. When the music comes on and the dancers engage, they enact, suggests Fuchs, the “spatiotemporal gestalt of the dance, which in turn draws them into its dynamics” (ibid). This entails a ‘mutual incorporation’ wherein each dancer incorporates the body of the other and body schemes extend and connect to form an overarching dynamic system (Fuchs and De Jaegher, 2009). Over time there emerges, from acts in which each partner learns to compensate for irregularities within their partner’s bodily comportments as directed toward the dance, what Fuchs calls a “harmonic, sinusoidal coordination of movements” (Fuchs, 2017a, p. 339). “Modifying Merleau-Ponty’s notion,” Fuchs continues, “we might speak of an operative we-intentionality, since for the skilled agents, the goal of the joint action is achieved through such habitual and largely prereflective bodily attunement” (ibid). And so, much like any individual’s ongoing action is constrained by a background of habitual dispositions and tendencies, the multi-agent system accrues a similar structuring, its actions proceeding according to comparable habitualities.

#### Moving Beyond Dyadic Body Memory

The conceptualization of dyadic body memory points in the right direction, that of widely distributed dynamics not exhausted through methodological individualism. However, we move beyond this account by explicating: how the enactive notion of autonomy helps reveal levels of cultural complexity – embodied in, for instance, a moment of dancing – that exceed the ‘sinusoidal coordination of movements’ and apply equally to less obviously ‘embodied’ gestalts’; how the patterns that comprise these interactive dynamics at shorter timescales (e.g., a ‘first’ dance between newlyweds) simultaneously borrow from and transform patterns that function at longer timescales within the socio-material niche (e.g., first dances on wedding days); how many of the norms of social interaction are embedded in trans-individual structures at multiple scales that work to sustain themselves as such; and, how the structural modifications that take place in social interaction continue to shape the sense-making of the individuals who comprise those interactions even when apart from them. Let us now consider an example that will help us to grasp the rich ecology of stabilities and evolving patterns that comprise any well-developed III.

Newly wedded P and S are moving their things to their new home together and must jointly load furniture into a removal van. When the couple come together to enact their identities as movers, they bring to the interaction a host of previously sedimented dynamics – stabilized in the context of their individual and shared concerns – that inform the activities of jointly lifting furniture into the back of the van. Thus, what emerges and stabilizes within the interaction is nested within dynamics of bodily capacities, but also of being newly wedded, of being in a relationship with particular role dynamics, of being in a particular culture in which marriage has a particular significance, and so on. In other words, what emerges as stable cannot be limited to an understanding of habitualities of the limbs (the ‘sinusoidal coordination of movements’); rather in stabilizing habitualities of the limbs, in sedimenting coordinated bodily dynamics, P and S produce and reproduce relatively autonomous structures at multiple scales with their own self-regulating norms, and in so doing also transform, however trivially, the larger habitus from which they borrow, e.g., how they enact their marriage feeds back into the habitus of marriage as enacted within their culture, and the sense of how it should be enacted. The ‘goal of the joint action’ for them is not only some task that specifically entails the coordination of joints and limbs, but also something akin to the maintenance of their interdependence; that is, enacting a concern for reproducing a kind of bond between each other and their socio-material milieu. We not only sediment ways of doing together, but rather, and more encompassingly, ways of being together. In other words, we are not simply enacting a joint procedural field, but rather, compatible interpersonal inter-identities that should be understood as constitutively dependent upon the socio-material constraints of the environment also, the meanings of which are transformed as they are introduced into social interaction.

The concept of III captures the right depth and width for a unit of analysis concerned with the socio-material complexity of human social individuation. Peering through the wide window offered by this unit of analysis, we observe a developmental whole comprised of the interdependent participation of various structuring patterns, each with unique life-cycles and spatio-temporal scales of transformation. On one end of the scale, short-lived patterns emerge and dissipate according to local constraints and contingencies of face-to-face interaction (think patterns of limbs lifting furniture together). On the opposite end of the scale, life long interpersonal relationships reshape a larger and publicly shared habitus (think marriages). The various life cycles and stages of such patterns may be seen as part of a coherent thread. This thread, which we identify as interpersonal inter-identity, need not be continuous in all its aspects, but just like a rope can be made up of multiple discontinuous pieces of fiber. In this way, there are patterns that live and die within the spatiotemporal horizon of a particular form of III, while there are other patterns that pre-exist and survive trans-generational change yet stay alive precisely by means of their integration within IIIs that characterize a multitude of interconnected social relationships.

From the example above we can start to see the different fibers that intertwine to form P and S’s thread of ‘being together,’ what their inter-identity is made up of. Dyadic body memory serves as a starting point for the analysis of this thread. It points to the visible and phenomenologically intuitive effect of a conservative tendency that both structures recurrent interpersonal encounters and is (re)structured by them. However, entangled with such dynamics are the norms – as instantiated in the signs and narratives of our cultures – that help us structure our interpersonal interactions and give them meaning, norms we borrow from a larger socio-material legacy but which are also transformed in our interactions to again become part of that legacy. Thus, what the individuation of IIIs points toward is not simply the sedimentation of patterns within the dyad or group, but also the processes by which the slowly-changing habitus are transformed within the dynamics of the interactions that comprise them.

Moreover, interpersonal relationships and the dynamics that support their successful enactments do not simply go dormant in the times between situations of face-to-face encounter. Clearly, in everyday life, people coordinate their behavior with respect to locally absent others. Romantic relationships offer rich examples of this phenomenon: persons ‘think’ and even dream of their loved ones, imagine activities for future encounters, invest time in the maintenance of shared homes, and generally behave with recourse to expectations about the continuation of relationships. Relationships, not only romantic ones, stay alive by alternating between dynamics of close range interaction and the dynamics of anticipation that constitute a continuing bond between persons. Indeed, our sense-making is constrained by the realities of such relationships even when we do not have some specific absent other in mind but encounter situations that reflect concerns that are relevant to the webs of interrelated patterns (IIIs) that characterize those relations. We return to this point later under the heading of trans-situational concerns.

We propose that the dyadic body memory underlying intercorporeal structures can be elaborated using the enactive notion of autonomy and the development of a notion of IIIs. Indeed, Fuchs himself writes that “intercorporeality… may also be regarded as an overarching system which over time gains its own pattern, autonomous dynamics and peculiar history” (2011, p. 205); and that, embodied interaction can “give rise to self-sustaining interaction patterns that go beyond the behavioral dispositions of isolated individuals. They may be attributed to a memory of the intercorporeal system and its partially autonomous dynamics …” (ibid, p. 206). Of course, there is an extensive body of literature pertaining to notions of social and/or ‘collective memory’ (e.g., Sutton, 2008; Wertsch, 2009; Michaelian and Sutton, 2017). However, the Fuchsian position is the first that we are aware of to point to the enactive notion of autonomy as a potentially central concept. Given the centrality of this concept to our account, we have chosen to use Fuchs as our leaving off point. That said, having developed our basic account from this new starting point, there will no doubt be much to be gained from future engagements with this body of work. The autonomous dynamics of the ‘overarching system’ we take up in the next section, suggesting how the dialectics of autonomy as articulated within recent enactive accounts provides a useful set of heuristics from which to begin our investigation into the processes of individuation responsible for the emergence of IIIs.

## Autonomy, Individuation, and Interpersonal Inter-Identities

### Autonomy as a Heuristic for Ongoing Individuation

**FIGURE 1:**
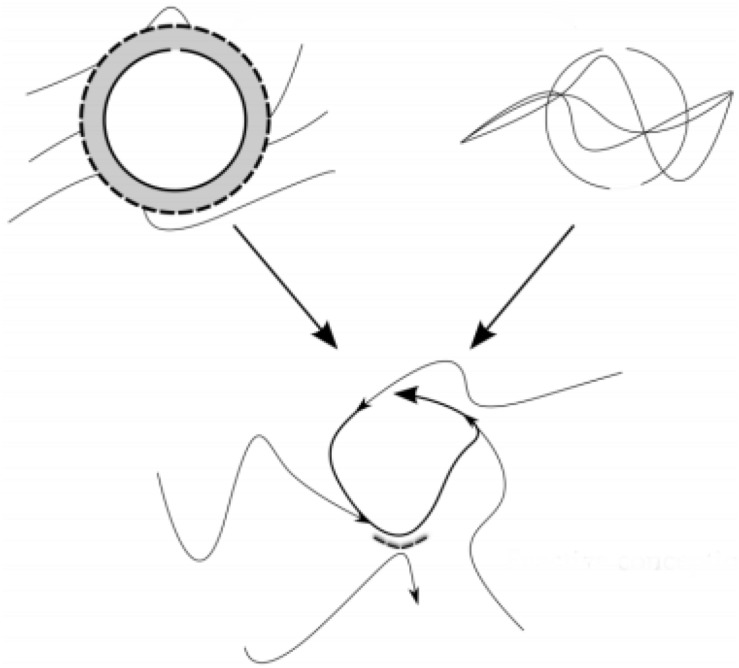
Self-production, represented by the graphic in the top right-hand corner, entails the effort to make oneself up from stuff available in one’s environment. Self-distinction, represented by the graphic in the top left-hand corner, entails closing oneself off from one’s environment. Held in dialectical tension over time, a dynamic represented by the graphic in the bottom, these dynamics provide the basis for the ongoing self-individuation of a given entity. Adapted and modified from Copyright Ezequiel Di Paolo 2015 as appears in Di Paolo (2018). This work is licensed under a Creative Commons Attribution-Non-Commercial-ShareAlike 3.0 Unported License.

In this section, we consider the enactive notion of autonomy as a primary heuristic for making intelligible some of the sociocultural processes relevant to the individuation of IIIs. This account is an elaboration of Fuchs’s notion of dyadic body memory, but also an effort to move beyond some of the limitations we see there. We begin by considering the dialectic account of autonomy as explicated by recent enactive accounts (Di Paolo et al., 2017, 2018), and then go on to suggest how this might inform our present position. These recent accounts offer a helpful characterization of autonomy, conceived of in terms of a temporally distributed dialectics between processes of self-production and processes of self-distinction responsible for the ongoing individuation of entities in a given domain. See Figure 1 for a graphic representation of these processes. Self-production, represented by the graphic in the top right-hand corner of Figure 1, describes an openness on behalf of a given entity to the flows of energy and matter available in one’s environment. Maximizing the dynamic of self-production means being totally open to all flows, as Di Paolo and colleagues put it, “the ideal condition for self-production would be one of total openness … [wherein] … every possible flow of matter and energy is taken advantage of” (2017, p. 133). But such a dynamic on its own would not facilitate individuation, for there would be no distinction of the entity from the environment. Self-distinction, on the other hand, represented by the graphic in the top left-hand corner of Figure 1, entails distinguishing oneself from one’s environment. An ideal realization of self-distinction would entail a relation of “total robustness to any environmental influence” (ibid). But again, if this were the only dynamic operative, individuation would be impossible, for self-production in any form ceases to be a possibility. Thus, in isolation neither dynamic is sufficient for individuation, for each in principle negates the other, however, when held in dialectical tension over time – a dynamic represented by the graphic in the bottom of Figure 1 – adaptively opening oneself up to or closing oneself off from this or that environmental condition (e.g., material flows, flows of energy, flows of information) provides the basis for the ongoing individuation of a given entity.

Self-individuating entities demonstrate conservation tendencies, motivating activities that preserve their individuation as such, both by continuing to be open to the necessary flows and inhibiting any inward flow that might disrupt or threaten them. Within enaction, we speak about the activities that support these self-regulatory processes in terms of sense-making. The classic example of autonomous entities are living entities such as cells, however, there are other examples of autonomously individuating entities, such as habits (Egbert and Barandiaran, 2014; Egbert and Canamero, 2014) and networks of habits or micro-identities (Varela, 1991; Barandiaran, 2017), social interactions (De Jaegher and Di Paolo, 2007; De Jaegher et al., 2010), and even, in some accounts, structures of communication (Luhmann, 1992). Of course, it is not obvious how a habit or network of habits can be considered an autonomous entity in the way that a cell can, and given the centrality of the notion to the account under development here, it is worth elaborating briefly on why habits can be considered as such.

The development of the notion of habit is a relatively recent one (Di Paolo, 2003; Barandiaran, 2008; Barandiaran and Di Paolo, 2014; Ramírez-Vizcaya and Froese, 2019) within enactive cognitive science, however, it is an important one for it provides a “blending category between the biological and the psychological,” and what Egbert and Barandiaran call “a theoretical building block for an organicist conception of mind” (2014, p. 2). Habits, within the enactive account, are self-sustaining behavioral structures that maintain their own organization through the behaviors they produce (Di Paolo, 2003), or, “self-sustaining patterns of sensorimotor coordination formed when the stability of a particular mode of sensorimotor engagement is dynamically coupled with the stability of the mechanisms that generate it, and which is reinforced through repetition” (Barandiaran, 2008). Habit in this account is taken as demonstrating the same circular self-production as other autonomous forms, e.g., autopoiesis. A single habit, contends Barandiaran, provides “a first analogy with life and a first approximation to a sensorimotor conception of identity and normativity,” whereby “through repetition … a habit can take on a life of its own: it is both the cause and the consequence of its own enactment” (2017, p. 13). It is worth noting, however, that habit is not merely another name for the autonomous organization found in the relations between neurodynamic patterns (or other supporting structures) and particular behaviors, rather, it develops these relations further by introducing the notion of plasticity, whereby repeating a particular sensorimotor correlation reinforces the organization that supports it, which in turn reinforces the probability of that correlation being enacted in self-similar conditions the next time around, evolving and shifting in response to the demands of its deployment. What emerges within such a dynamical organization, within the habit, is a very minimal sense of identity, a focal point concerned with its own maintenance. And, given that any habit relies on certain conditions – rate of repetition, particular environmental structures etc. – boundaries of viability are enacted, stipulating certain conditions as required if the habit is to be kept alive, i.e., the norms of its own self-regulation (Barandiaran, 2017).

Within the enactive account, however, we also move beyond a single self-reinforcing habit to *networks of habits*. For Barandiaran the habit network is partly meshed within the brain, where much of the plasticity and selection lies, and, within a relatively complex brain the self-maintenance of habits needn’t be reduced to mere recurrent self-reinforcement but might rely on more “relationally complex, interdependent architectures” (ibid, p. 14) The general contention is this, if the network’s plastic interconnectedness is complex enough sensorimotor regulations will engender large scale equilibrating tensions within the network, whereby “sensorimotor compensations … take place to maintain the capacity of the agent to keep behaving coherently” (2017, p. 14). In other words, when the network has gained sufficient complexity, it’s self-conservation becomes its basic operational norm and it is motivated to act in ways that sustain its identity as such.

In what follows we suggest that habits and networks of habits are also operative in the relationship between recurring social interactions and their broader ecologies, and indeed, it is such entities and the relationships between them that make up the more encompassing entities that are IIIs (the ropes that bind the threads together).

### Autonomy in the Socio-Material Domain

De Jaegher and Di Paolo write that a “history of coordination demarcates the interaction as an identifiable pattern with its own internal structure” (2007, p. 492). This “identifiable pattern” we can consider an individuating entity in the relational domain, a transient autonomous identity manifest for the duration of the enactment of the social interaction. However, it is the more fine-grained ‘structuring’ of this pattern that concerns us when speaking about IIIs, particularly as interactions become recurrent. Such structuring, we contend, is best thought about in terms of the coenhabiting of spatiotemporally distributed entities that shape the activity of those who enact them, whilst borrowing from and transforming a larger socio-material niche. In considering this account, however, it will be helpful for us to first reflect on the notion of autonomy as it applies to the social domain.

Reflecting upon the self-organized emergent order that characterizes his home life, author Karl Ove Knausgaard writes “If this didn’t happen on its own, at least it occurred without planning, and through all the 1000s of small daily adjustments that were made in order to make everything flow as easily and effortlessly as possible, patterns were created, eddies, ways of being, both in the children and in the parents” (2018, p. 35). Making intelligible these patterns, these eddies and ways of being; articulating the interbodily dynamics that underwrite these and the other examples we have been considering, we must first briefly consider the notion of autonomy within enactive approaches to understanding social phenomena. De Jaegher and Di Paolo (2007) contend that a very general form of autonomous organization emerges in any social interaction. When we coordinate our behaviors in interaction, the emergent dynamics dispose the interactors to sustain, modify, or terminate their encounter. The transient autonomous entity that is the social interaction thus instantiates a form of operational closure, wherein operations within the system relate to the perpetuation of other processes within the system in a closed-loop. This entity, the social interaction, De Jaegher and Di Paolo characterize in terms of “the regulated coupling between at least two autonomous agents, where the regulation is aimed at aspects of the coupling itself so that it constitutes an emergent autonomous organization in the domain of relational dynamics” (2007, p. 493)^2^. In acknowledging such autonomy we also recognize that such systems can sustain themselves beyond the concerns of their individual components, e.g., a conversation that persists despite neither party really desiring it to. These interactions cannot be reduced to the actions or intentions of either individual, but rather they install a “relational domain with its own properties that constrains and modulates individual behavior” (De Jaegher and Di Paolo, 2007, p. 494).

Some social interactions take on a historical dimension — i.e., sustained or recurring interactions — and take shape according to the coordinations, breakdowns and recoveries that constitute their history. As suggested also in the dyadic body memory account, these histories empower interactors to more easily coordinate ongoing interactions and recover from breakdowns. De Jaegher and Di Paolo also note that “we often perceive some interactions as improving over time, and recovery from a break down as a sort of learning” (2007, p. 496). Recall Knausgaard observation above about the patterns and ways of being that emerged “through all the 1000s of small daily adjustments that were made in order to make everything flow as easily and effortlessly as possible” (2018, p. 35). Learning happens in such interactions at multiple levels simultaneously. If we return to the earlier newlyweds example, P learns how to lift the chair with S who is much smaller than her and they come to habitually adopt that mode under such conditions; but the autonomous relational system P-S also learns to self-regulate under particular conditions so as to maintain its autonomy as such, and thereafter works to pull P and S into self-similar configurations under self-similar conditions. Through repeated interactions under varying conditions, a whole repertoire of self-regulating dynamics sediment in the relational system until what emerges is a network of more or less stable inter-regulating patterns. Such patterns demonstrate conservation tendencies as the norms of their own self-regulation, motivating activities that sustain their organization as such, pulling interactants into modes of being, often experienced as a kind of ‘falling into.’ This account differs from – though is perfectly congruent with – the account of participatory sense-making developed by De Jaegher and Di Paolo (2007), in so far as it acknowledges not just the emergence of a basic autonomous dynamic in social interaction, but the emergence of more fine-grained autonomous structures within the interaction, structures which are likely to facilitate the more general autonomy of the interaction, but need not act in this way. They are *likely to* largely because behaviors that maintain interactions have more opportunity to stabilize than those that lead to breakdowns; they *need not* facilitate the autonomy of the social interaction if is recurrent enough despite the breakdowns, e.g., a couple who have the habit of getting into heated arguments that instantly flair up and lead to breakdowns of the general autonomous dynamics of the social interaction.

When we consider the emergence of IIIs within interaction, we do so through the explication of a couple of related concepts (1) the notion of coenhabiting: a set of processes wherein the interdependencies that undergird the autonomous structures comprising IIIs are established within a given socio-material niche, whilst also transforming that niche at longer timescales; and (2) the notion of a co-optimal grip: a social extension of the notion of optimal grip – proposed by Bruineberg and Rietveld (2014) – in which living entities tend toward a more optimal relationship to their environment given their situated concerns. In the sections that follow we develop these concepts and their relations in some detail.

## Coenhabiting and the Co-Optimal Grip

### Optimal Grip and Enhabiting Autonomous Identities

The tendency toward an optimal grip, as revitalized by Bruineberg and Rietveld (2014), is a sui generis form of intentionality that describes the tendencies of skilled human agents to strive for a better grip on their present situation by reducing ‘disattunements’ – experienced as ‘deviations from an optimum’ or ‘tensions to be reduced’ – between endogenous and exogenous dynamics (2014, p. 3). Illustrative examples might include adjusting your distance to someone ahead of you in a cue, finding just the right spot from which to regard a painting, or settling into position when taking a snooker shot. Depending upon the present concerns and abilities of the acting embodied subject, the environment will be encountered in ways that afford or ‘solicit’ some actions and not others, experienced in the form of tensions to be reduced. Such solicitations are said to be supported on the organism side of things by what Bruineberg and Rietveld – leaning on the work of Frijda et al. (1989) – refer to as patterns of *action readiness*, i.e., bodily states that exist somewhere between abilities and actual actions (2014). Thus, an organism tending toward optimal grip is constantly responding to solicitations, and, in so doing, re-organizing their patterns of action readiness, which in turn open up additional solicitations, which if acted upon lead to novel states of action readiness, and so on. Patterns of action readiness imply what Bruineberg and Rietveld term a ‘selective openness,’ such that when the embodied subject is organized according to some particular pattern of action readiness they experience pronounced sensitivities to certain features (extrinsic norms, signs, shapes, sounds, etc.) of their environment, and by implication limited sensitivities to other features. In acknowledging these dynamics, we can get a sense for how the autonomous dialectics described by enactive accounts show up in the perception and action of embodied subjects and how existing autonomous organizations can maintain their organization over time. And so, here we draw a parallel between the ‘selective openness’ that arises in the relationship between patterns of action readiness and particular environmental conditions, and the autonomous dialectics between self-production and self-distinction. Selective openness suggests something of a boundary in our attention and peripheral awareness. For instance, as well as moving toward certain features of my environment (including other social agents) and opening myself to their effects (acts of self self-production), I am equally as likely to retract from other features of my environment, or dampen their possible effects (acts of self-distinction). I am open, but selectively so. Not incidentally, the kinds of dynamics implied here are congruent with Kyselo enactive account of the ongoing individuation of the self (which is always-already social) when she writes about it as emerging *through and from a world* (Kyselo, 2014, p. 8). It is both dependent upon or *participating* with certain features of the world (self-production), whilst also emancipating itself from it by making *distinctions* (self-distinction). Part of what we are doing here is refining this language by suggesting that when tending toward an optimal grip, these dynamics of individuation manifest in the perception-action cycles of embodied subjects as patterns of selective openness, our attention being actively drawn to that which is relevant to the reproduction of the autonomous dynamics organizing attention in the first instance. Here then, we can say that the autonomous dialectics apply to the entity as a whole (i.e., brain-body-environment or multiple-brain-multiple-body-environment systems), but selective openness characterizes the means by which they show up within the perception and action of the subjects that are at their center. In sum, selective openness helps realize the general operative dynamics of multiple autonomous ecological entities acting according to the norms of their own self-regulation. Under this reading, “deviations from an optimum” can be seen as perturbations to the relatively sedimented autonomous dynamics that support ongoing individuation. And so, responding to such deviations is acting according to the self-regulatory norms of these entities, e.g., habits and networks of habits at various timescales. Here the notion of optimal grip quite straight-forwardly parallels the notion of sense-making, as it serves the ongoing regulation of some existing autonomous structure.

However, existing self-regulatory norms are not always adequate to situational demands, or indeed, norms motivated by structures at different timescales may be in some tension with each other. In such instances there may be no obvious ideal or optimal to return to, and thus, sense-making, understood as “the capacity of organisms to perceive their external environments according to norms … and to act according to these norms in a way that continually affirms and even strengthens the probability of their ongoing existence,” is not adequate (Weinbaum and Veitas, 2017), for it presupposes the autonomous structure that generates the norms in the first instance, and does not adequately account for its emergence^3^. It is here, then, that we must introduce the notion of enhabiting. Absent the guidance of the norms of previously existing autonomous structures, when tending toward an optimal grip, there is a more general situationally relevant norm at play, i.e., to establish an optimal position to one’s situation from which to act. Driven by such a norm – a kind of metastability seeking – we suggest that previously incompatible organizations can resolve into novel integrated organizations (Scott, 2014), bringing forth novel interdependencies between bodily and environmental structures, and facilitating the emergence of new self-regulating wholes. Such events are what we hope to capture in the notion of enhabiting. Selective openness then, is not limited to the self-regulating norms of existing autonomous organizations, but can also support the emergence of novel organizations: I am selectively open to that which serves the ongoing individuation of existing habits (sense-making), but also to that which serves the emergence of novel habits or the integration of existing ones into novel ones given situational demands (enhabiting).

### Enhabiting the Pressure Passer

Consider the processes of enhabiting a ‘personal identity’ as a particular kind of Brazilian Jiu-Jitsu (BJJ) practitioner. Within BJJ the permutations of positions and strategies are vast and the practitioner cannot hope to develop proficiency in them all. This is understood by coaches. Thus, as well as demonstrating technique, their job at longer timescales is one of assisting the coachee in ‘finding their game,’ i.e., the set of proficiencies well-suited to their natural attributes. This process of finding and later refining one’s game can be viewed through the lens of enhabiting.

When first entering the gym, the ‘selective openness’ characterizing the *absolute beginner* – given their prior individuation as someone who enters unfamiliar communities of practice – is attuned to solicitations relevant to their immediate concern of finding their place in the group. They will be selectively open to, for instance, hierarchies of authority, permissible, and impermissible ways of comporting oneself, sartorial norms, and norms about how to receive instruction. An optimal grip at this point primarily pertains to finding a place from which to take up their position as a learner. Sensitivities to the details of the technique are not yet well-developed, however, iteratively responding to solicitations engendered by particular modes of selective openness; with time one transcends their identity as an *absolute beginner*, transitioning to a *novice learner.* Now, although sensitivities to the norms previously mentioned persist and continue to constrain activity, the acquiring of technique becomes the trainee’s primary concern. The dynamics of enhabiting are already at play here, however, the transition from *novice learner* to *pressure passer* will help us articulate them in detail, as this provides a more circumscribed set of processes for consideration.

For the first year or so as a *novice learner*, the typical coaching is to remain as open as possible to all the moves demonstrated. There are many reasons for this: for instance, it gives the novice learner enough time to get a feel for the primary positions and acquire some defensive and offensive options from them (e.g., from the ‘back,’ from the ‘mount,’ from ‘side control’); it also gives the learner the opportunity to discover what is well-suited to their natural attributes, personality, etc. Thus, at this stage – in broad strokes – we can say that the novice is selectively open to *as much technique as possible*; reflected, for instance, in their taking notes on *all* the technique demonstrated after each class. Sensitivities at this point tend to be to the coarse-grained dynamics of the movements, analogous to the novice guitar player who moves from one chord to another, but is not yet introducing flourishes into their transitions.

For those wishing to progress past the stage of novice learner, this mode of openness becomes somewhat problematic. Spreading their practice time across as much technique as possible, the practitioner can never hope to acquire any real depth of knowledge. By now, however, continually tending toward an optimal grip during practice and in conversations with coaches and training partners, when watching instructionals, and watching footage of professional fighters with similar attributes, the learner is developing sensitivities such that a certain ‘path’ of development solicits: one set of “tensions to be reduced” comprise solicitations of a more encompassing set over longer timescales. This is more commonly spoken about in terms of the emergence of a ‘game.’

As a novice learner, the norms that maintain the identity of our learner as a capable person are in tension both with his identity as a good student and the existing sensorimotor norms that organize the coordination of his muscles. Tending toward optimal grip, actions that best satisfy this stack of norms give rise to interdependencies that undergird novel, though, at this stage, relatively diffuse organizations, e.g., the habits and networks that support basic techniques. Our protagonist is a larger male who lacks the dexterity of his smaller and more athletic training partners. In the process of acquiring basic techniques some have a kind of stickiness which collectively suggest that he can use his weight and size to his advantage by maintaining top position. Working from these positions he is selectively open to opportunities to leverage them further and he begins to identify with them. Encountering the so-called *pressure passing* style, something like a game, a more integrated network of moves that work well together in a particular situation, begins to solicit. The sense of *identification with* grows, and the solicitations promise to resolve some lingering tensions. A new set of norms emerge pertaining to the pursuit of a particular path of development.

Having the physical attributes that he does, this proves a fruitful path for our learner, and his additional attention to its details leads to increased success in sparring. Now, he is selectively open to what might advance his developmental path further still and thus he becomes differentially sensitive to the affordances that reflect that path, whilst others lose their glow^4^. This implies a multi-scale process, dependent upon both local/situational solicitations, and solicitations at longer timescales (van Dijk and Rietveld, 2017). Welcoming completely novel environmental structures into our personal umwelts, or transforming the relations between structures already therein, such moments signal the integration of some previously diffuse or even disparate organizations into more integrated wholes and can have enduring transformations on what we are selectively open to in any relevant situation. Only in this context do the finer details of our learners ‘game’ begin to cohere, for now what he is selectively open to has been reduced from *every bit of instruction in every class* to the *instruction that will help develop ‘my’ game*. Here, he is undergoing a more holistic process of individuation, such that a relatively invariant domain specific autonomous whole emerges – a personal identity – with its own self-regulating norms and dynamics of selective openness, i.e., *me as a pressure passer*.

What we are describing are nested processes of enhabiting at multiple timescales in the context of a set of evolving and overlapping concerns^5^. To enhabit then, is to individuate, it is to construct through iterative processes of tending toward an optimal grip, identities that we not only bring into being through our activities, but identities we thereafter live within. In enhabiting, by manifesting novel structural interdependencies between body and environment, we transform impersonal potentialities into meaningful relations through which we make sense of our on-going experience.

### Co-optimal Grip and Coenhabiting Interpersonal Inter-Identities

Being together implies an expansion to the degrees of freedom of the individual embodied subject, there is a lot more that can be done in orchestration with others. But this also expands the horizons of uncertainty; by multiplying the capacity and diversity of collaborative work we also expand potential sources of dis-attunement. This expansion, however, is counterposed by the incorporation of trans-interpersonal regularities and constraints available in the socio-material niche. The circular generative processes that characterize these transformations – which depend upon the regulation of processes of interaffectivity, joint action, and joint attention – we refer to in terms of coenhabiting. These are processes in which we are jointly “laying down a path in walking” (Maturana and Varela, 1987; Thompson, 2007). Importantly, following Stapleton and Froese (2015), we are not making claims here regarding the emergence of a collective subject, if understood to be a kind of collective first-person. Rather, we conceive of IIIs as entailing collective second-person perspectives, which can imply the realization of shared lived perspectives (ibid, p. 232)^6^. We agree with these authors that genuine collective subjectivity requires tight material integrity, a requirement that only multicellular bodies have. However, the shared lived perspectives characteristic of collective second-persons can be derived from the behavioral and affective integrity of social interactions, particularly as they become recurrent and sediment into compatible IIIs. In social interactions, the increase in tensions to be reduced relates in large part to the coordination of multiple nested self-regulating norms. In reality, any abstraction from the near-infinite number of self-regulating norms enacted in any embodied social interaction is going to be insufficient. Nevertheless, it seems there are some norms most of us most of the time are guided by when acting together. Here we abstract a couple of such norms as basic forms of concern present in most social interactions and we use them as a kind of prism through which to refract the processes of socio-material individuation (i) a general concern to “get along” (longer timescale); and (ii) a concern for “successfully acting together” (shorter timescale). The co-regulatory behaviors of interactants that allow them to maintain these concerns within what we might call their viability limits (experienced as forms of interactive stability or flow) can be seen as being shaped by what we refer to as a tendency toward co-optimal grip. Tending toward co-optimal grip, however, is not limited to the re-realization of existing concerns but can drive the emergence of novel concerns also. We will explore these ideas in more detail below. Achieving and/or maintaining interactive stability requires interactants being selectively open to features of the interaction itself and the normatively rich environment in which it is taking place. This necessitates those involved operating from what we might call states of sympathetic readiness. This can be supported by acting in accordance with basic co-available norms; for example, successfully ‘getting along,’ and ‘successfully acting together.’ However, much as in the individual case, they must also be sensitive to the solicitations that will better serve their integration and the emergence of novel shared organizations and their attendant self-generating norms. Here we can talk about the whole multiple-brain-multiple-body-environment system as enabling patterns of ‘selective openness’ in which the coupled interactants each demonstrate an openness or receptivity to certain features of their environments and effectively ignore or dampen the effects of others. Over time, such processes allow for the simultaneous gearing of individual participants into dyads and groups; and the gearing of dyads and groups into their broader socio-material milieus. Each component coenhabits synergistic interdependencies with the others comprising the larger whole and their respective environments; both transforming and being transformed by the larger whole in the process. However, they also enact distinctions from these larger wholes, thus participating in the coenhabiting of autonomous structures at multiple scales simultaneously. These processes motivated largely by a general tendency toward co-optimal grip – which we will develop in some detail now – support the emergence of autonomous socio-material structures from simple social habits and networks of habits to more encompassing IIIs.

#### Coenhabiting the Drilling Pair

We return to the domain of Brazilian Jiu-Jitsu. Our protagonists this time are two female competitors. We start the account where the primary concern is already successfully acting together. It is common in BJJ for the coach to demonstrate a particular technique using a subject picked from the coachees present, moving through the sequence a number of times, each iteration adding details or emphasizing some aspect. In so doing, they provide a set of co-available constraints with which coachees coordinate their drilling together. As well as coordinating according to the constraints supplied, successfully acting together and maintaining the ‘drilling’ dynamic depends upon both training partners being selectively open to (i) both intra and interbodily dynamics, such as, physical capacities, bodily dimensions, relative skill levels; and (ii) relevant environmental features such as available space on the mats, implicit norms of the gym, the time allotted for drilling etc. Being together under such conditions (ideally) takes the form of both partners acting together to assist in one another’s reproducing the instructions of the coach. Here we introduce the notion of a co-optimal grip.

In this example, co-optimal grip can take on a rather literal interpretation. For instance, when the ‘passive’ partner assists the ‘active’ partner to gain the optimal position – such as a grip on a lapel – so as to efficiently reproduce the technique. Enacting such a grip, interactants not only tend toward an optimal grip with respect to some shared concern but co-regulate their activities so as to enable optimality in their partner’s efforts also. This co-optimal grip when drilling within the general concern of successfully acting together is felt by our pair as an efficiency (a kind of shared flow) in the application of the technique under situational demands.

Throughout the actual drilling scenario, the dynamics of ‘getting along,’ on the other hand, manifest in a general care that training partners have for one another, and maintaining a co-optimal grip with respect to this often requires explicitly checking in. Although varying across gyms, drilling is typically initiated by a collective hand clap along the lines “Everybody got that? OK, 1, 2, collective clap,” after which pairs peel off^7^. When partners pair up they do not simply start drilling, but rather introduce themselves and shake hands (at least this is common practice in many Western gyms) if they have not met before, or maybe share some pleasantries if they have. Either way, just prior to drilling they will engage a ritualistic and ubiquitous hand-clap-fist-bump.

Although there is no striking allowed in most BJJ, there is significant bodily contact, each partner striving for control over the other’s body so as to be able to gain a submission, all the while being challenged with the full resistance of their opponent. One might speculate, given the intimacy of the sport, the ubiquity of the hand-clap-fist-bump helps initiate bodily contact in a way that frames what follows in terms of a general dynamic of comradery (such gestures are also ubiquitous before and after sparring), motivated by the concern to ‘get along.’ Tending toward a co-optimal grip throughout, drilling partners check in with one another as they go, indicating, often with grunts and hisses, if someone is being a bit heavy handed or less than cooperative. Anything that might threaten the dynamic of getting along is made up for with an additional hand-clap-fist-bump before returning to drilling. Gross deviations from optimal generate feelings of awkwardness, of shame or embarrassment, and require efforts from both parties to make right. If, for instance, one partner injures the other whilst being over-zealous, recovering the dynamics of ‘getting along’ relies as much upon the injured party’s graciousness in accommodating the apologies of the injurer as it does upon their displays of shame and making apologies.

Interestingly then, any activity at the shorter timescale of ‘acting successfully together’ unfolds against the background of ‘getting along’ and derives at least some of its meaning and normative value from such a framing. However, it also feeds back into it. What it means to “get along” is reciprocally entangled with what it means to “successfully act together.” Indeed, the norms of the gym described in the earlier example are also continuing to shape action and they also maintain such reciprocal dependencies. When tending toward a co-optimal grip all of these elements are simultaneously at play. Consequently, one might speculate, coenhabiting interdependencies is all the more probable to the degree that tendings toward co-optimal grip satisfy these nested concerns. In other words, if the manner in which the drilling partners carry out their drill also satisfies their concerns of getting along, the norms of the gym, and the intrabodily norms of the individual interactants, the pattern is more likely to be coenhabited than if it only satisfied one or another concern.

But tensions and incompatibilities are almost constant in social interaction. What we observe then, much as we observe in the individual case, is that when existing norms do not suffice for the ongoing regulation of the interaction, by maintaining the general dynamic of tending toward a co-optimal grip – a kind of social metastability seeking – novel more integrated organizations can emerge. Such dynamics become obvious, when, for instance, our training partners meet outside of the gym and the norms of their IIIs as sedimented in the gym during practice do not suffice to meet the demands of the situation. Indeed, the often rather humorous disattunements inspired by such instances are illustrative of the various normative dimensions of social interaction, dependent, as they are, both upon regulating with respect to existing autonomous structures and situationally tending toward co-optimal grip. For instance, you meet your colleague whom you have only ever interacted with in the seminar room by the fridges in the supermarket, and ‘fall into’ a conversation about philosophy that seems at odds with the situated norms of your interaction. In such instances, the self-generating norms of the previously sedimented structures that normally pull you into felicitous interactions do shape the interaction, but they prove insufficient and must be informed by the more situated norms characteristic of tending toward co-optimal grip. Such dynamics are always operative within recurrent interactions, we simply don’t notice them for the majority of our interactions, with people with whom we have not built up highly flexible repertoires of socially coordinating, occur within self-similar situations. We typically encounter our training partners at training, our colleagues at work, our house mates at home. Thus, our falling into particular modes of interaction are typically experienced as relatively well attuned to the environments in which they are occurring.

Being open to the features that maintain the interactional dynamics of *getting along* and *successfully acting together* also means being closed, in effect, to the myriad of other elements that the dyad could, in theory, be paying attention to, e.g., the mild injury one has in their knee; what their training partners are doing on the mats around them, the noises coming from outside the gym, etc. In other words, ongoing individuation at this level too depends upon the dialectics of self-production and self-distinction. Through their utterances, gestures, and the myriad ways they comport themselves when tending toward co-optimal grip, social interactants exhibit boundaries in the dynamics of their perceiving and acting that limit or possibly even dampen the potential effects of certain environmental features. In the BJJ case, this might show up initially as simply not paying attention to anything but the features relevant to the concerns we have spoken about. However, when interactions are prolonged, or when they become recurrent, the results from these processes become more pronounced. In our example, this initially evinces in the training partners focusing on some co-available feature of their interaction in defiance of their coach’s instructions, but as sessions iterate our pair work out a specific template that best supports their learning. With each iteration, and ongoing tendencies toward co-optimal grip, this basic pattern becomes more stable and more refined, coming to function like a template of interrelated anticipations and arches of action that acquire a degree of portability. Now, when they drill collar chokes instead of arm locks, they follow more or less the same template.

At a certain point, the template effectively disappears into the background like a mutually available but silent groove that acts as the backing for ongoing improvisations. Now, ongoing instructions from the coach about how to sequence the drill might be completely ignored, the patterns themselves emerging as wholes and leveraging the activities of their components in service of their reproduction. If, for instance, instruction is given to the class to go easy on a drill, the dyad that has coenhabited their own routine may fail in some genuine sense to even hear the instruction, simply falling into their previously sedimented patterns. In other words, the patterns come to organize the dyad as such, readying the interactants for certain kinds of collaborative acts under certain conditions, disposing them to be open to some features of their environments and effectively closed off from others.

Although we do not have the space to elaborate it much here, there is an interesting inter-regulatory relationship that exists between various autonomous patterns that emerge in social interactions. Take for instance the autonomous pattern comprising a particular network of social habits and the general autonomous pattern that is the social interaction. Not only do particular networks serve to shape the interaction according to particular norms, they also, typically, serve to maintain the dynamic integrity of social interactions on the whole. Indeed, when pulled into social interactions, particularly as they have taken on the feature of recurrence, such a pull is made all the more felicitous by the habits and networks we have established previously. In other words, the patterns we coenhabit take on a co-constitutive relationship with the basic pull to coordinate characteristic of social interaction. In this way, the recurrent autonomous social interactions generate and help maintain the various structures that comprise IIIs, and vice-versa. We might think of it like this, when interactions become recurrent we experience not just a pull to coordinate, but a pull into normatively infused patterns of coordination that facilitate ongoing coordinations, patterns infused with the coenhabited outcomes of previous interactions under self-similar conditions.

In summary then, through iterative and nested processes of tending toward co-optimal grip, inter- bodily dynamics, entangled with particular environmental features, sediment as autonomous ecological entities at multiple timescales, engendering relatively invariant patterns of selective openness that our training partners fall into during self-similar interactions. In short, what we are describing is the coenhabiting of interpersonal inter-identities that serve as the backgrounds within which we participate to make sense together, backgrounds which function a bit like a silent rhythm section that lays down a groove for us to either rehearse our well-worn tunes together or break out in improvisation, sometimes even changing up the groove in the process. Much like autonomously organized identities in other domains, such entities manifest norms of their own self-regulation. Consequently, when animated by such entities, acting in ways that do not accord with such norms are experienced as “deviations from an optimum,” thus soliciting actions that reproduce themselves as such. In this manner, we are lived through by such entities. Our individual tendencies toward an optimal grip and our capacities for habituation allow us to gear into patterns much larger than ourselves and thereafter act on their behalf, even when finding our own personal identities within them.

## Trans-Situational Concerns

Previous sections have considered cases in which processes of (co)enhabiting both give rise to habituated identities at the individual level and interpersonal inter-identities in recurrent real-time reciprocal interactions, however, there are some corollary cases that we wish to briefly point to now, i.e., how concerns engendered as part of the IIIs that have arisen in real-time reciprocal interactions with others might contribute to the sense-making of the individuals who comprise those relationships, even when apart from such interactions.

In interaction with others wherein we engender IIIs, we coenhabit tendencies and capacities that are relevant to the maintenance of those interactions and the satisfaction of concerns that are present therein; we get a feel for the ‘games’ we are involved in and stabilize the skills necessary to play, or develop them further. If I am part of a community of Theravada Buddhist practitioners, in interaction with others in that community I am organized for interactions with them, which implies that I adopt concerns that are not unlike theirs in some key respects and stabilize ways of acting in relation to them (Loaiza, 2019); indeed, it is such shared concerns and acting in relation to them at multiple timescales that allow us to refer to ourselves collectively as Theravada practitioners.

These tendencies and their attendant bodily capacities are substantially grounded in the interdependencies between the bodily and environmental structures wherein they come into being, however, much of the value of such tendencies and capacities to me as an individual is that they can be enacted outside of their specific contexts, and thus, we recognize in them a degree of *portability* (Cuffari et al., 2015; Di Paolo et al., 2018). For instance, the bodily and environmental structures that undergird my capacities as a Theravada practitioner and the concerns they reflect, will be borrowed from during the enactment of my emerging interpersonal inter-identities if I find myself in the company of a community of Mahayana Buddhists. My new beginning is not always a radically new one^8^. Given that the Mahayana community shares many concerns with the Theravada community, finding my place in the new community is bootstrapped on my having found my place in comparable communities previously, the emerging interdependencies between bodily and environmental structures borrowing from existing dynamics first sedimented elsewhere. However, this kind of portability, we suggest, does not pertain solely to situations of real-time reciprocal interactions, but can also apply to situations in which, for instance, one merely anticipates the presence of another.

Consider another excerpt from Knausgaard, when he reflects autobiographically about his preparation to host his older brother and his brother’s friend in his new flat. He recalls the activities he underwent, all the while tending toward an optimal grip:

“I stood by the door and tried to see the room through Yngve’s and Asbjørn’s eyes. The typewriter on the desk, that looked good. The poster of the barn and bright yellow corn under the dramatic black American sky, that was good, a source of inspiration. The poster of John Lennon, (…) And my record collection on the floor against the wall, it was large and impressive, even for Asbjørn, who I was told knew what he was talking about. On the downside, the book collection was limited, comprising only 17 volumes, and I didn’t have enough experience of other collections to determine what impression the various titles made. Beatles and The Snails by Saabye Christensen couldn’t be too far wide of the mark though. The same was true for Ingvar Ambjørnsen. I had three of his books. I left Novel with Cocaine open on the table and placed a couple of issues of Vinduet next to it, one open, one closed. Three books open seemed a bit much, it looked arranged, but no one would be suspicious of two open and one closed, that was perfect” (2016, p. 45)

This passage suggests something about what is entailed in tending toward an optimal grip in a socially relevant situation even when not in real-time interaction with others it might concern. Knausgaard evokes an identity in his imagination; overlaying it upon the scene it engenders a constellation of tensions to be reduced. In Knausgaard example, his imaginings pertain to his imagined self as Yngve’s younger writer brother and his desire to gear into the world Yngve and his friend represent. The intricacies of such imaginal identities will not bother us here, however, it seems uncontroversial to claim that such an identity, whatever its explication, evinces concerns at least partially coenhabited in relationship with Yvnge, and an individual concern to individuate within the sociol structures Yvnge and his friend represent.

Of course Knausgaard cannot know what his visitors’ reactions will be and must rely upon reducing any disattunements engendered as he moves about making sense of the scene. But from where do the bodily structures that underwrite such disattunements come? A reasonable supposition seems to be that they are substantially those that also undergird the enactment of the IIIs to which they pertain. We are changed in our interactions with others, such that even when we decouple from them we do so in ways in which their concerns continue to shape our individual actions. Much as with concerns and attendant actions in the transition between Theravada and Mahayana communities, there appears to be a kind of portability here also, but here it is to situations that only virtually reflect something about the original relation. In the example above, Knausgaard sense-making is shaped by concerns originally stabilized in relationship with his brother and the socio-material milieu they collectively integrate with and transform when coenhabiting their IIIs, and what shows up as relevant in his environment is precisely that which allows him to continue that process.

One of the more possibly illuminating illustrations of such integrations is the example of someone purchasing an item of clothing. Our clothes are very often our first (re)introduction to others and can help establish the basis for certain types of coordination, whereby wearing one item of clothing or another can signal probabilities of being organized according to certain concerns within a given sociocultural milieu. Thus, whether conscious of it or not, our preference for some piece of clothing over another can be thought about in terms of a function of our tending toward an optimal grip when organized by an individual concern to synergistically integrate with a particular group/collective/other. The experience of preferring *just that pair of shoes* being also part of the dynamics that serve the (re)individuation as a component of that larger system.

If our individual concerns to integrate with particular social systems are central enough, they become interdependent with the concerns of that system such that even when apart from others with whom we comprise such systems, when encountering situations that are relevant to our collective concerns we are likely to act in ways that are congruent with them. Moreover, when we don’t act in manners that are congruent we are likely to experience some degree of disattunement, thus soliciting congruent actions, and in so doing inviting us to reproduce the socio-material order and its specific concerns, or to enhabit new ways of being that reflect our individuation in relationship to these larger structures. In the cases above it might be clearer which relationships inform which activities (e.g., it is primarily Knausgaard relationship with his brother that informs his activities when arranging his room; one’s desire to be part of the biker gang informs their decision to purchase the leather jacket), however, our sense-making predominantly operates within concerns sedimented in the coenhabiting of IIIs most of which are subtle and not as easily exemplified as our above examples (e.g., relationships with early caregivers, parents, significant others, colleagues and peers). In these ways, the social mind inexorably infuses the individual mind, and vice versa, and we must acknowledge any pure disentangling as utterly impossible.

Moreover, IIIs typically arise in the presence of institutional and cultural constraints and in so doing effectively act as components in the production and reproduction of those larger social entities. As we have already suggested, any particular individual will be a component in many such entities. But interestingly, the inverse relationship is also true, as much as any individual is but a component in the social whole, any social entity is but a component in the individual whole, indeed the individual is in fact a composite of the vestiges of many such social entities, who lie in wait for their reproduction in the furnace of some future social interaction. In sum, as much as we live within the multiple patterns that we coenhabit with others in our socio-material niches, we are equally lived within by them, we are animated by them. We are, in short, multiply animated.

An obvious corollary of this is that it makes little sense to speak of a unified, coherent self, and rather, the individual person, the embodied subject, is, in fact, an entanglement of personal and interpersonal inter-identities that take shape in the presence of certain conditions and certain others, and leave their dynamical traces and their attendant concerns to contribute to the whole in their absence. Such identities are not wholly distinct but are overlapping, interpenetrating, and inter-regulating and are brought into conversation with each other in situations that solicit more than one particular identity and its attendant capacities.

## Conclusion

In the final sections we arrived at the idea that perhaps much of what characterizes individual sense-making at the personal level and outside of social interactions can be understood as the manifestation of concerns inextricably linked to our histories of acting together, i.e., as members of families, relationships, institutions, communities of practice, etc. From an experiential point of view, this equates with the felt sense of the relative stability and continuity of our personal lives across situations, particular groups of people, life contingencies, and distinctions between public and private spheres. In the account we have presented, this phenomenon points to the emergence and stability of interpersonal inter-identities. Persons not only show a spontaneous tendency to re-enact styles of bodily action in coordination with others with whom they have a history together, they also manifest stable and socially grounded dynamics that sediment in the longer/slower timescales. Consequently, we have developed an account of interpersonal identity that is not exhausted by instances of direct interpersonal interaction, – such as in participatory sense-making – and an attendant dyadic body memory. As such, we have tackled questions regarding the particularities of long-term histories of social interaction and illuminated some of the dynamics underlying the normative dimensions of social life.

Starting with the individual case we formulated the relationship between tending toward an optimal grip and the processes of enhabiting responsible for establishing interdependencies between bodily and environmental structures, which thereafter comprise the habits and networks of habits of individuals in their particular niche. We then extended these insights to the social domain. Working with the assumption that individuals very often encounter one another with already existing compatible concerns for ‘getting along’ and ‘successfully acting together,’ we formulated an account of how when acting according to these concerns and a general tendency toward a co-optimal grip, we can resolve incompatibilities and tensions in situated interaction into relatively stable, though ever evolving, patterns of being (or becoming) together at multiple timescales, from simple social habits, such as the coordination of limbs while lifting furniture; to more complex networks of habits, such as those that organize routines between training partners, or, indeed, those that characterize romantic relationships. We also suggested that in coenhabiting these novel patterns we reproduce or transform (however trivially) the trans-individual habitus wherein they come into being. Froese has recently suggested that the “formation of a genuinely collective social memory only requires that people are creatures of habit” (2018, p. 1). The account of IIIs developed here puts some meat on the bones of that claim. The ‘genuinely collective social memory’ might be envisaged like an ever-evolving collection of mutually supporting nets. Each net comprises a habitus, and each lattice of ropes the interpersonal inter-identities that characterize our social relationships, with all their individual yarns, and fibers, and intricate interdependencies facilitating their messy integration with the whole.

## Author Contributions

MJ and JL contributed to both he conception and execution of the present manuscript revision, read and approved the submitted version. Although the ideas contained therein are largely derived from MJ’s Ph.D. work, at every step of the process JL contributed to their placement and refinement within the present text. MJ wrote the bulk of the text, however, JL also contributed written sections to the manuscript.

## Conflict of Interest

The authors declare that the research was conducted in the absence of any commercial or financial relationships that could be construed as a potential conflict of interest.

## Footnotes

1 There are empirically documented examples of more complex and spatiotemporally distributed patterns that nevertheless betray the same kinds of invariances, for instance, the strategies that long term running partners enact when traversing difficult paths (Allen-Collinson and Hockey, 2017).

2 Much empirical work highlights a tendency for individuals in interaction to couple through their spontaneous coordination (e.g., Shockley et al., 2003; Richardson et al., 2007; Davis et al., 2016), and for autonomous dynamics to be at work in even the most minimal of embodied interactions (e.g., Auvray et al., 2009; Froese and Paolo, 2010).

3 This limitation in the notion of sense-making has been pointed out by other authors also. Beaton (2014), for instance, asks “how can nonsense ever become sense for us, if perception only ever presents the world within the existing structures of our understanding?” (p. 153). See also James (in press) for an extended consideration of the limitations with the notion of sense-making and a more indepth treatment of the notion of enhabiting.

4 Being animated so has the effect of engendering a selective openness in how one enacts their world, meaning that they will be open to certain environmental features, whilst appearing to dampen any potential influence of others, potentially making sensitivities to environmental features that do not serve the self-generated norms difficult or even impossible. The pressure passer, for instance, experiencing strong identifications with other pressure passers and receptive to participating with them, whilst being veritably insensible to someone extolling the virtues of the ‘leg lock’ game and maintaining strong distinctions in relationship to them. Infants, from as early as 5 months old (Marno et al., 2016), selectively attend to utterances from native speakers of their language, even showing a preference for learning from such speakers, suggesting a possible inhibitory effect mediating their response to non-native speakers.

5 Importantly, these processes extend beyond the bounds of the gym and might also emerge during anticipatory acts, for instance, acts of thinking, imagination, languaging, e.g., when rehearsing particular moves and sequences of moves when lying in bed; or talking with a peer about the various aspects of ‘your game.’

6 See said paper for a rich empirically informed discussion of these matters.

7 The call and response within which the clap is enacted might be thought about as an instance of joint speech (Cummins, 2019) embedded in a social habit operative at a very short timescale.

8 A related notion is observed by Merleau-Ponty also, when he writes about how a skilled organist can easily transition between organs without having to start again entirely from scratch (Mooney, 2012, pp. 146–147). Of course, it is not a direct translation into the domain of social interaction, but it seems uncontroversial to suggest that there are some parallels here.
